# Risk factors and mitigating measures associated with bile duct injury during cholecystectomy: meta-analysis

**DOI:** 10.1093/bjsopen/zraf076

**Published:** 2025-08-02

**Authors:** Rowan Burns, Katie L Connor, Rachel V Guest, Chris C Johnston, Ewen M Harrison, Stephen J Wigmore, Ahmed E Sherif

**Affiliations:** Department of Clinical Surgery, University of Edinburgh, Royal Infirmary of Edinburgh, UK; Department of Clinical Surgery, University of Edinburgh, Royal Infirmary of Edinburgh, UK; Department of Clinical Surgery, University of Edinburgh, Royal Infirmary of Edinburgh, UK; Department of Clinical Surgery, University of Edinburgh, Royal Infirmary of Edinburgh, UK; Scottish Liver Transplant Unit, Edinburgh Transplant Centre, Royal Infirmary of Edinburgh, UK; Department of Clinical Surgery, University of Edinburgh, Royal Infirmary of Edinburgh, UK; Department of Clinical Surgery, University of Edinburgh, Royal Infirmary of Edinburgh, UK; Department of Clinical Surgery, University of Edinburgh, Royal Infirmary of Edinburgh, UK; Scottish Liver Transplant Unit, Edinburgh Transplant Centre, Royal Infirmary of Edinburgh, UK; Department of Hepatobiliary and Pancreatic Surgery, National Liver Institute, Menoufia University, Shibin Elkom, Egypt

## Abstract

**Background:**

Cholecystectomy is a common procedure with a notable risk of iatrogenic bile duct injury. Understanding the factors contributing to bile duct injury and the effectiveness of preventative measures is crucial for improving surgical outcomes. This meta-analysis aimed to identify and synthesize high-quality evidence on risk factors and mitigating measures associated with bile duct injury after cholecystectomy.

**Methods:**

Following the PRISMA guidelines, a comprehensive literature search was conducted across multiple databases. Included studies reported on adult patients undergoing cholecystectomy with relevant risk factors for bile duct injury. Meta-analyses of unadjusted and adjusted risk estimates were conducted with a random-effects model to account for heterogeneity. The study period across all included studies spanned from 1989 to 2016.

**Results:**

The review included 31 studies comprising 6 513 599 cholecystectomies and 18 259 bile duct injuries. The primary risk factors identified were male sex (adjusted odds ratio 1.27, 95% confidence interval 1.13 to 1.39) and acute cholecystitis (adjusted odds ratio 1.74, 1.27 to 2.39). The critical view of safety was inconsistently documented and not statistically linked to reduced bile duct injury. Intraoperative cholangiogram's routine use did not show a statistically significant association with reduced incidence of bile duct injury (adjusted odds ratio 0.92, 0.70 to 1.23).

**Conclusion:**

Male sex and acute cholecystitis significantly increase the risk of bile duct injury after cholecystectomy. Risk stratification for these patients before surgery would ultimately aid the shared decision-making consent process.

## Teaser

This meta-analysis examined risk factors and preventive measures for bile duct injury (BDI) following cholecystectomy. The results, based on 31 studies involving over 6.5 million surgeries, identified male sex and acute cholecystitis as significant risk factors. The study suggests that preoperative risk stratification could help improve decision-making and potentially reduce the risk of BDI.

## Introduction

Cholecystectomy to treat gallstone diseases is one of the most commonly performed operations worldwide, with approximately 50 000 cholecystectomies performed annually in the UK and 800 000 in the USA^1,2^. Nevertheless, detrimental outcomes related to injury to surrounding structures remain not uncommon. Iatrogenic bile duct injury (BDI), with a reported incidence of 0.2–1.5%, remains the most clinically significant morbidity associated with up to a three-fold increase in mortality, long-term negative impact on quality of life and the cost burden it imposes on healthcare systems^3–6^.

The level and extent of BDI and its association with concomitant vascular injuries directly impact patient management. Multiple classifications have been used to define and describe the level of BDI. Strasberg’s^7^ and Bismuth–Corlette^8^ classifications are the most commonly used by tertiary hepatobiliary centres managing those complex injuries worldwide.

Multiple non-modifiable factors, such as surgery for acute cholecystitis (AC), patient morbid obesity and male sex, have been associated with increased incidence of BDI. They are thought to contribute to the increased difficulty of the surgery, whether laparoscopic or open. The introduction of the critical view of safety (CVS) principle, is believed to have contributed to a lower rate of BDI by reducing misidentification of the common bile duct as the cystic duct or cystic artery^7,9^. The routine or selective use of intraoperative cholangiography (IOC) and other intraoperative imaging as mitigating measures were proven helpful in identifying bile duct stones, anatomical biliary variations and in recognizing BDI. Nevertheless, neither routine nor selective use of IOC has been shown to reduce the risk of BDI in randomized clinical trials^9–12^. Human factors relating to operating surgeon experience, visual perception illusion and lack of corrective feedback have been modestly investigated^13,14^.

Therefore, this review aimed to systematically review the literature and synthesize better-quality evidence on the association of potential risk factors and mitigating measures with BDIs after cholecystectomy.

## Methods

The systematic review was conducted and reported following the PRISMA guidelines^15^. The research methodology and protocol were prospectively registered on the International Prospective Register of Systematic Reviews (PROSPERO) registration number CRD42020177318 (https://www.crd.york.ac.uk/prospero/).

### Search strategy

A literature search was conducted of the MEDLINE, EMBASE, Scopus, Cochrane Central, and Web of Science databases and a grey literature search of the ProQuest database. Full search terms for each database are presented in *Table S1*. Studies were included from the inception of each database to May 2023.

Two independent reviewers (R.B. and A.E.S.) screened the titles and abstracts of the pooled search results for relevance, as per inclusion and exclusion criteria, using the Rayyan online screening tool^16^. Full-text articles of the first screening results were obtained. A second screening of full-text articles was performed to determine the final included studies.

### Study population and outcomes

The main purpose of this study was to identify risk factors and preventive measures with good-quality evidence supporting their association with iatrogenic BDI after cholecystectomy. The primary outcome measurement was the odds ratio (OR) of BDI incidence. The study population included adult patients who had undergone cholecystectomy, laparoscopic or open, and for whom exposure to relevant risk factors had been recorded.

The primary outcome, the OR of BDI incidence, was chosen to provide focus and clarity in this meta-analysis. Unlike confirmatory trials, where multiplicity requires adjustments, such concerns are typically not applicable to observational studies. Instead, robustness is ensured through predefined criteria, quality assessments, and sensitivity analyses.

### Inclusion and exclusion criteria

Prospective or retrospective observational studies that included patients over 18 years of age were considered eligible. Studies were included in the meta-analysis if they contained data from which it was possible to calculate a risk estimate. Full-text publications in the English language only were included. Studies were excluded if their population underwent alternative methods of cholecystectomy, such as robotic, subtotal, or single incision cholecystectomy, or if they consisted of patients presenting with BDI as this prevented analysis of potential risk factors. For studies with an overlap in data, the study with the most recent time frame was included. BDI was defined as any injury to the biliary tract sustained during cholecystectomy and manifested by symptoms and signs of biliary obstruction, leak, or stricture. Different classifications of the type and level of BDI were reported, including Bismuth and Strasberg classifications.

### Data extraction and risk of bias assessment

As the outcome of interest was the incidence of BDI in relation to the underlying risk factors, the factors were included in the quantitative analysis if at least three studies provided suitable data to be pooled, where populations analysed were similar. Other factors included having undergone qualitative analysis. The variables extracted were study characteristics, population, settings, primary endpoints, BDI incidence and definition, all risk factors included in the study analysis, variables used in the multivariable analysis if applicable, significant variables in the analysis, and the number of events and OR for each risk factor reported in the analysis. Data from univariable analyses were recorded as unadjusted risk estimates, and from multivariable analyses were recorded as adjusted risk estimates. If risk estimates were not reported in the primary data, these were manually calculated using the event rate numbers reported.

The included observational studies were analysed for risk of bias according to the Newcastle–Ottawa scale^17^. Furthermore, the quality of the studies was assessed as either high, medium or low, according to the principles laid out by the Cochrane guidance for risk of bias evaluation^18^.

### Statistical analysis

The meta-analysis was performed using Review Manager (RevMan) version 5.3, The Cochrane Collaboration, 2020. Meta-analysis was performed for both pooled unadjusted and adjusted risk estimates separately, and they were presented on individual forest plots. Unadjusted risk estimates for the outcomes of interest were pooled together using the Mantel–Haenszel statistical method for dichotomous data, whereas adjusted risk estimates were pooled with the inverse variance method. On the basis that individual studies were likely to be measuring different effect sizes, a random-effects model was employed throughout. Heterogeneity was measured by estimating study variance (τ^2^) using the DerSimonian–Lair estimator and the Cochrane Q test, based on the χ^2^ test. The percentage total variance was calculated to generate the *I*^2^ statistic, which was considered significant at > 50%. Potential clinical and statistical causes of heterogeneity were explored. An outlier analysis was performed, and where the 95% confidence interval (c.i.) of the study failed to cross the 95% c.i. of the pooled OR, the study was removed for that risk factor. A sensitivity analysis and leave-one-out analysis were performed to examine the influence of individual studies. Using the Baujat method, studies that contributed significant heterogeneity but had little effect on the risk factor were removed.

The validity of the results in this meta-analysis was enhanced by carefully addressing the methodological challenges of combining population-based and observational studies from different populations, which limit their reproducibility and interpretability. The methodology used to address these challenges was restricting the suitability of pooling studies according to studies with a clear and consistent definition of BDI after cholecystectomy, an objective demonstration of acceptable quality and low risk of bias using a qualitative risk of bias assessment tool, an adjustment of potentially confounding competing risks using multivariable analysis, and with a low statistical heterogeneity after sensitivity analysis. When the former conditions were met, statistically significant risk estimates were considered more valid.

## Results

**Fig. 1:**
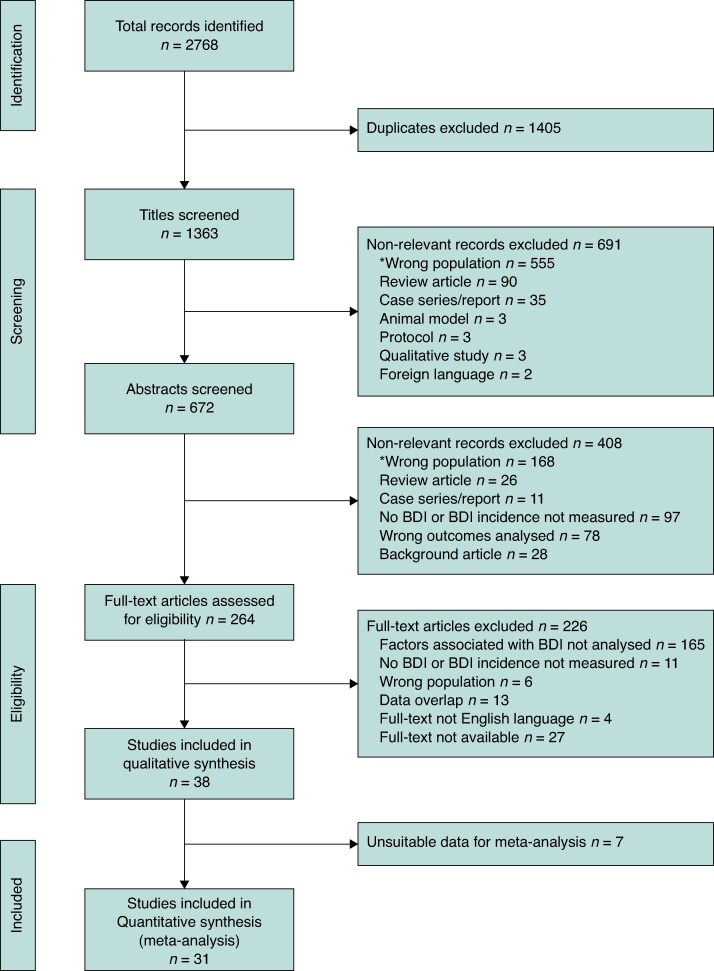
PRISMA flow diagram demonstrating the details of studies screened, included, and excluded *Wrong population is defined as studies that included a population of patients with BDIs and did not include the comparator population without BDIs. BDI, bile duct injury.

The literature search identified 2786 records, of which 672 were assessed for eligibility after the first screening. Out of the 672 full-text studies evaluated, 31 studies with 6 513 599 cholecystectomies and 18 259 BDIs met the inclusion criteria with suitable data and underwent quantitative synthesis. The characteristics and risk of bias assessment of these studies^4,11,13,19–45^ are demonstrated in *Table 1*. Four additional papers were included as part of the qualitative analysis. The full details of inclusion and exclusion are illustrated in a PRISMA flow diagram (*Fig. 1*). Of all included studies, 15 collated data from national, state-wide, or registry databases, whereas 16 studies collected data from single institutions^48^.

**Table 1:** Characteristics of included studies and risk of bias assessment

	Study Period	Study design	Population Description	Population	Country	BDI incidence	Outcome measurement	Quality assessment score		
								NOS Selection (of 4)	NOS Comparability (of 2)	NOS Outcome (of 3)
Altieri *et al*. (2017)^45^	2000–2014	Retrospective cohort	Patients undergoing LC for benign biliary disease	391 945	USA	0.12%	Incidence of CBD injury	4	1	3
Aziz *et al*. (2015)^19^	2010–2012	Retrospective cohort	Patients undergoing LC for GS disease	39 476	USA	2.57%	Incidence of BDI	4	2	1
Beliaev *et al*. (2016)^20^	2004–2009	Retrospective cohort	Patients undergoing LC after undergoing ERCP for clearance of CBD stones	183	New Zealand	3.28%	Incidence of major BDI	4	2	3
Buddingh *et al*. (2011)^21^	2004–2009	Retrospective cohort	Patients undergoing OC/LC for GS disease	856	Netherlands	3.04%	Incidence of BDI	3	2	3
Diamantis *et al*. (2005)^22^	1991- 2001	Retrospective cohort	Patients receiving cholecystectomy for symptomatic gallstones	3637	Greece	0.52%	Incidence of BDI	3	1	1
Dolan *et al*. (2005)^23^	1990–2000	Retrospective cohort	Patients undergoing LC	2 841 186	USA	0.16%	Incidence of BDI	4	2	3
El-Dhuwaib *et al*. (2016)^13^	2001–2013	Retrospective cohort	Patients undergoing LC for benign biliary disease excluding where there is a stone in the BD	572 223	UK	0.09%	Incidence of BDI requiring reconstruction	4	2	3
Fletcher *et al*. (1999)^24^	1988–1994	Retrospective cohort	Patients undergoing OC or LC for benign biliary disease	20 084	Australia	0.22%	Incidence of BDI	4	2	3
Flum *et al*. (2003)^46^	1992–1999	Retrospective cohort	Patients undergoing OC/LC for benign biliary disease	1 570 361	USA	0.50%	Incidence of BDI	4	2	3
Georgiades *et al*. (2008)^25^	1993–2005	Retrospective cohort	Patients undergoing LC	2184	Greece	0.69%	Incidence of BDI	3	2	3
Giger *et al*. (2011)^11^	1995–2005	Retrospective cohort	Patients undergoing LC for benign biliary disease	31 838	Switzerland	0.32%	Incidence of BDI	4	2	1
Grönroos *et al*. (2003)^26^	1995–2002	Retrospective cohort	Patients undergoing LC	3736	Finland	0.86%	Incidence of BDI	4	1	1
Hobbs *et al*. (2006)^27^	1980–1999	Retrospective cohort	Patients undergoing cholecystectomy for biliary disease	33 309	Australia	0.23%	Incidence of BDI	4	2	3
Kamran *et al*. (2013)^28^	2008–2010	Retrospective cohort	Patients undergoing LC for benign biliary disease excluding those with AC or previous abdominal surgery	2061	Pakistan	0.82%	Incidence of biliary injury	3	2	3
Kholdebarin *et al*. (2008)^29^	1991–1997	Case-control	Patients with BDI after LC	Not reported	Canada			2	2	2
Kiviluoto *et al*. (2001)^30^	1995–1996	Randomized clinical trial	Patients undergoing open *versus* lap cholecystectomy for AC	63	Finland	0%	Hospital mortality and morbidity (including incidence of BDI)	N/A	N/A	N/A
Kohn *et al*. (2018)^31^	2009–2015	Retrospective cohort	Patients undergoing LC for GS disease	800	USA	0.88%	Incidence of BDI	4	1	1
Krähenbühl *et al*. (2001)^32^	1995–1997	Retrospective cohort	Patients undergoing LC for GS disease	12 111	Switzerland	0.30%	Incidence of BDI	3	2	3
Kum *et al*. (1996)^33^	1989–1991	Retrospective cohort	Patients undergoing LC for biliary disease	478	Germany	0.84%	Incidence of BDI	4	0	2
Lilley *et al*. (2017)^34^	2004–2010	Retrospective cohort	Patients > 66 undergoing OC/LC for biliary disease—excluding those with neoplasms ordinary fistula	472 367	USA	0.30%	Incidence of CBD injury	4	2	2
Mangieri *et al*. (2019)^35^	2012–2016	Retrospective cohort	Patients undergoing LC for benign biliary disease	217 775	USA	0.20%	Incidence of BDI	4	0	1
Natsume *et al*. (2017)^36^	2006–2012	Retrospective cohort	Patients undergoing OC/LC for benign biliary disease	1289	Japan	0.62%	Incidence of BDI	4	2	1
Ragulin-Coyne *et al*. (2012)^37^	2004–2009	Retrospective cohort	Patients undergoing OC/LC for benign biliary disease	111 815	USA	0.26%	Incidence of BDI	4	2	1
Russell *et al*. (1996)^38^	1990–1993	Retrospective cohort	Patients undergoing OC/LC for benign biliary disease—excluding those with previous abdominal surgery	30 211	USA	0.16%	Incidence of major BDI	3	1	3
Shawhan *et al*. (2015)^39^	2004–2011	Retrospective cohort	Patients undergoing OC/LC with IOC for benign biliary disease	96	USA	4.17%	Incidence of bile leak	4	2	1
Sheffield *et al*. (2013)^47^	2000–2009	Retrospective cohort	Patients who underwent cholecystectomy for biliary colic/biliary dyskinesia, AC, or chronic cholecystitis	92 932	USA	0.30%	Incidence of biliary reconstruction claims within one year	4	2	2
Söderlund *et al*. (2005)^40^	1999- 2003	Prospective cohort	Patients undergoing LC	1563	Sweden	1.54%	Incidence of BDI	4	1	3
Taragarona *et al*. (1998)^41^	1977–1996	Retrospective cohort	Patients undergoing LC for biliary disease	4612	Spain	0.69%	Incidence of BDI	4	0	1
Tornqvist *et al*. (2015)^42^	2005–2010	Retrospective cohort	Patients undergoing LC for GS disease	50 041	Sweden	1.46%	Incidence of BDI	4	2	3
Tornqvist *et al*. (2016)^43^	1990–2005	Case-control	Patients with BDI after LC	Not reported	Sweden			4	2	3
Yaghoubian *et al*. (2008)^44^	2000–2006	Retrospective cohort	Patients undergoing LC for benign biliary disease	2470	USA	0.77%	Incidence of BDI	4	2	1

BDI, bile duct injury; NOS, Newcastle-Ottawa score; LC, laparoscopic cholecystectomy; CBD, common bile duct; GS, gallstones; ERCP, endoscopic retrograde cholangiopancreatography; OC, open cholecystectomy; IOC, intraoperative cholangiography.

After data extraction, variables hypothesized to increase the risk of BDI that had suitable data for the meta-analysis were male sex, AC as an indication for surgery, and low surgeon experience (< 100 cases performed). Mitigating factors against the risk of BDI, such as adopting a CVS before cystic duct ligation and the routine use of intraoperative cholangiography, were analysed. Despite the definition of BDI in the included studies showing a degree of variation, the majority of included studies utilized well-recognized classifications in describing and grading the severity of BDIs. Full details on the definition of BDI and the results of other risk factors analysed (patient age and comorbidities, and laparoscopic *versus* open surgery) are in the *supplementary materials*.

### Male sex

**Fig. 2:**
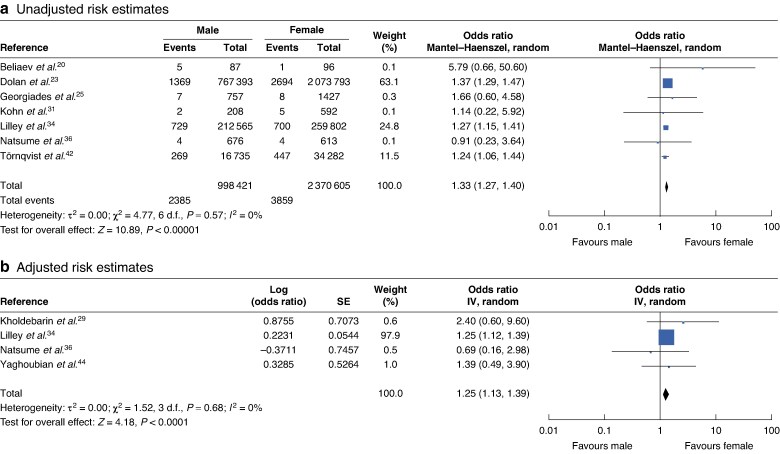
Forest plots showing pooled unadjusted and adjusted risk estimates of patient sex association with the incidence of bile duct injury **a** unadjusted and **b** adjusted risk estimates. d.f., degrees of freedom. IV, inverse variance; SE, standard error.

Twenty papers investigated the relationship between male sex and BDI. After sensitivity analysis, 9 of these had suitable data for pooling. The pooled OR (95% c.i.) for unadjusted and adjusted data were 1.33 (1.26 to 1.40) and 1.27 (1.13 to 1.39), respectively (*Fig. 2*). One study by Kamran *et al*.^28^, using multivariable analysis, found that male patients were more likely to have severe gall bladder inflammation, a longer intraoperative time, higher chances of conversion from laparoscopic to open cholecystectomy due to obscure Calot’s triangle or adhesions, and subsequently increased risk of BDI. Kum *et al*.^33^ shared a similar finding from their univariable analysis. Three studies^28,37,44^ showed male sex was associated with a statistically significant increase in the risk of all-cause complications.

### AC

**Fig. 3:**
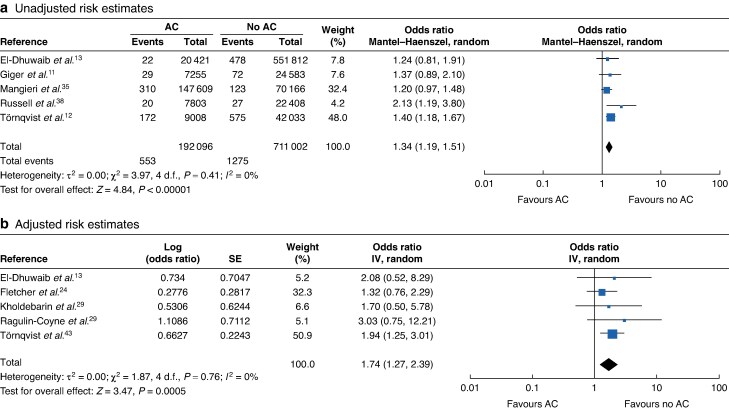
Forest plots showing pooled unadjusted and adjusted risk estimates of the impact of AC indication on the incidence of bile duct injury **a** unadjusted and **b** adjusted risk estimates. d.f., degrees of freedom; AC, acute cholecystitis; IV, inverse variance; SE, standard error.

A total of 17 studies explored the correlation between AC and BDI, 9 of which had data suitable for pooling with no statistical heterogeneity and had information on the definition of AC. The pooled OR (95% c.i.) for unadjusted and adjusted data were 1.33 (1.26 to 1.40) and 1.27 (1.13 to 1.39), respectively (*Fig. 3*). All included studies demonstrated that AC was associated with an increased risk of BDI. The majority of included studies defined AC based on clinical diagnosis (full details are in the *supplementary materials*). Only one study^49^ utilized the 2012 revised Tokyo Guidelines for AC, and the authors found that the severity of inflammation was correlated with BDI risk, with more severe AC (Tokyo grade II and III) being linked to a more considerable risk of BDI^43^. Two papers investigated the link between inflammation and BDI. One^25^ showed that the presence of any inflammation incurred a greater risk of BDI (OR 3.61, 95% c.i. 1.27 to 10.21), whereas another^36^ showed a link between a high white blood cell count and BDI.

### Surgeon experience

Ten papers^11,13,25,27,32,35,36,41,42,46^ analysed the association between the surgeon’s experience and BDI incidence. Due to the variability in reporting the surgeon’s experience, risk estimates from only 3 papers^11,25,32^ were pooled, and the total number of operations performed by the surgeon were dichotomized into groups of < 100 and > 100 operations (*Fig. S1*). The pooled OR 2.48 (95% c.i. 0.27 to 22.7) had significant heterogeneity and a too-wide 95% c.i. El Dhuwaib *et al*.^13^, Hobbs *et al*.^27^, and Flum *et al*.^46^ found a declining BDI risk associated with increased surgeon yearly case volume. El Dhuwaib *et al*. showed that, where a surgeon had performed more than 80 cholecystectomies, the risk of BDI significantly decreased in comparison with surgeons who had performed fewer than 20 (OR 0.56, 95% c.i. 0.39 to 0.80). An analysis of the United States National Surgical Quality Improvement Program registry (2012–2016)^35^ concluded that resident teaching cases were protective against BDI (relative risk 0.56, *P* = < 0.001).

### CVS and aberrant anatomy

Four studies examined CVS as a widely adopted intraoperative approach for BDI prevention. Avgerinos *et al*.^50^ and Manatakis *et al*.^51^ compared CVS with other dissection approaches. Despite the prevalence of CVS utilization in their cohort, the incidence of BDI in both groups was too low to detect a significant difference. Kohn *et al*.^31^ showed that only 12.4% of the operation notes described CVS correctly in detail consistent with the definition by Strasberg^7^ and they did not find a statistically significant association with the incidence of BDI. Gimenez *et al*.^52^, enquiring about surgeons’ common practice of laparoscopic cholecystectomy, found that only 21.8% of 446 respondents had correctly identified the elements of CVS.

One study^36^ explored the impact of aberrant biliary anatomy on BDI. The authors retrospectively analysed the magnetic resonance cholangiography imaging of 1289 patients. They reported aberrant bile duct anatomy as an independent predictor of BDI during cholecystectomy (OR 16.56, 95% c.i. 3.28 to 83.51).

### IOC

**Fig. 4:**
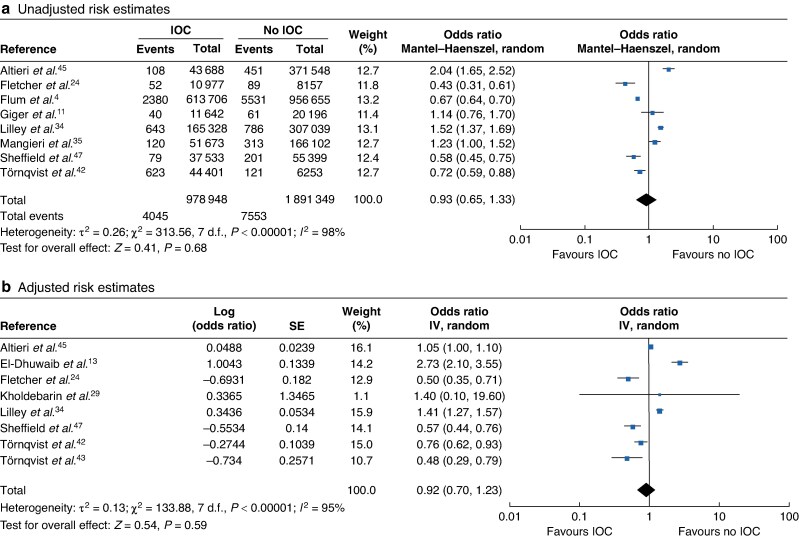
Forest plots showing pooled unadjusted and adjusted risk estimates of the impact of IOC utility on the incidence of bile duct injury **a** unadjusted and **b** adjusted risk estimates. d.f., degrees of freedom; IOC, intraoperative cholangiography.

IOC use during cholecystectomy was analysed by 15 studies, 11^11,13,24,29,34,35,42,43,45–47^ of which had suitable data for meta-analysis, as demonstrated in *Fig. 4*. Included studies reported data comparing IOC *versus* interventions without IOC, whereas studies that compared routine with selective use of IOC were excluded. The unadjusted pooled analysis did not show that routine IOC use with cholecystectomy had a statistically significant association with a reduced incidence of BDI (OR 0.93, 95% c.i. 0.65 to 1.33). Both adjusted and unadjusted pooled analyses were significantly limited by the heterogeneity of the included studies, which could not be improved with sensitivity analysis.

Several population-based studies^24,42,46,47^ showed an associated increased risk of BDI after cholecystectomy when IOC was not used. Sheffield *et al*.^47^ reported that, despite the standard risk-adjustment methods (multivariable logistic regression model) showing a statistically significant increased risk of BDI in the non-IOC group, an instrumental variable level analysis, controlling for the unmeasured confounding of the variability of hospitals’ and surgeons’ IOC use, demonstrated that the association was not statistically significant. Tornqvist *et al*.^42^ found that IOC was shown to be protective against BDI in patients with a history or presentation of AC undergoing surgery.

Other studies^13,34,45^ showed contradictory associations between the increased incidence of BDI and IOC use. The association was hypothesized not to have been causal. However, this likely reflected the utilization of IOC in higher-risk patients or as a confirmatory test when BDI was identified during an operation.

## Discussion

This study analysed a total of 6.5 million cholecystectomies, and an incidence of BDIs ranging between 0.1 and 4.1% was found. The analysis aimed to focus on putative risk factors to improve risk stratification for patients undergoing cholecystectomies. An evidence gap in the literature is reflected in the liberal use of expert opinions in the latest Safe Cholecystectomy Multi-Society Practice Guidelines recommendations^3^. Of all risk factors or mitigating measures analysed, male sex and AC were the non-modifiable risk factors identified to have a statistically significant association with BDI.

Risk stratification before surgery ultimately aids the shared decision-making consent process. Factors such as male sex, obesity, AC, and previous abdominal surgery are commonly considered risk factors for difficult cholecystectomy and subsequently the risk of BDI^9,11,25,28,36,43,53^. The evidence synthesized from this meta-analysis on male sex and AC risk factors will help to inform the consent process when such patients attend surgery. Risk stratification in these situations can also help with resource allocation, the degree of expertise, and support the operating surgeon may require in high-risk patients.

Despite some reports^35^ showing that more experienced surgeons had better results, another analysis^13^ showed that cholecystectomy performed by medically qualified residents in teaching hospitals resulted in lower rates of BDIs. Despite CVS being a strongly advocated approach to identify anatomical variation and minimize the risk of BDIs^3,9,53^, the operating surgeon’s perception of how much dissection is required to achieve CVS is variable. A previous analysis^31^ reported that only 1 in 5 of surgeons’ operation notes accurately described the CVS. On the other hand, human factors such as visual perceptual illusion, external stressing factors, and burn-out and fatigue can significantly contribute to misperception errors leading to BDI. Further research on cognitive psychology and human factors is required to guide surgeons in improving their insight and corrective feedback in stressful conditions^14^.

The utilization of imaging modalities during cholecystectomy, such as IOC, to reduce the risk of BDI remains contentious. In this meta-analysis, despite measures to combat heterogeneity, the results of pooling adjusted risk estimates showed significant statistical heterogeneity. Several studies^42,45,47^ in the analysis showed some benefit in certain situations, whereas others showed no such benefit. In studies showing an increased association between IOC use and BDI, the role of IOC in the delineation of the biliary anatomy as a confirmatory tool for immediate detection of suspected BDI should be considered over causality^34^. These results are in line with previous systematic reviews^3,54,55^ that could not provide good evidence to recommend routine or selective IOC as a BDI prevention modality. One recent meta-analysis^10^ comparing the routine *versus* selective use of IOC has suggested that routine use of IOC could help reduce the risk of BDI and be proven cost-effective. The safe cholecystectomy multi-society guidelines^9^ could only conditionally recommend the use of intraoperative imaging in patients with a history of concurrent AC.

The surgical approach for cholecystectomy this study analysed was laparoscopic compared with open surgery. Including studies from the inception of databases inevitably introduces confounding due to the evolution of the laparoscopic approach and the progression of surgeons’ learning curves over time. Some recent studies showed that open cholecystectomies are associated with more risk of BDI, but it could be confounded by selection bias because laparoscopic converted to open or upfront open cholecystectomies are commonly reserved for the most challenging ones^36,42^. Other surgical approaches, such as subtotal cholecystectomy or robotic-assisted approach, were beyond the scope of this review and were part of the exclusion criteria.

A key limitation of this meta-analysis is that the risk factors identified for BDI are non-modifiable. Despite the efforts made to tackle the methodological challenges of meta-analysing observational studies, the analyses of BDI preventive measures were ultimately limited by the significant heterogeneity among the included papers, which impacted the ability to provide recommendations with reasonable strength and certainty. Whereas future studies using more homogeneous data sets may help identify the actual effect of modifiable risk factors. Using the best available evidence from diverse sources or expert opinions remains a more pragmatic approach, allowing clinicians to recognize high-risk cases and apply tailored intraoperative safety measures to mitigate BDI risk^9^.

Meta-bias arising from the inter-variability across the included populations and over-interpretation of pooled statistically significant risk estimates is a recognized challenge in meta-analysing observational studies^56,57^. Although sensitivity analysis is a valuable tool in eliminating heterogeneous studies, it could skew the overall effect size, and it does carry risks of potentially inflicting selection and/or attrition meta-bias.

Another limitation of this study is the inability to quantitatively analyse the impact of time from symptom onset to intervention on the risk of BDI because of the inconsistency in defining the timing of emergency cholecystectomy of the index admission. Although the safe cholecystectomy multi-society guidelines^9^ recommended surgery within 72 hours from the onset of symptoms for patients with mild AC, future studies with standardized reporting of symptom duration and operative timing are needed to clarify the relationship between delayed intervention and the risk of BDI.

Most data in this meta-analysis come from high-income countries, with limited reporting from low- and middle-income countries (LMICs), potentially underestimating the global burden of BDIs. Establishing national and international cholecystectomy registries with mandated reporting could improve data accuracy, enhance patient safety, and facilitate the equitable adoption of advanced surgical techniques, such as robotic cholecystectomy, in LMICs^58,59^.

The future direction for bile duct damage prevention after cholecystectomy should be focused on preoperative risk stratification and prediction to allow appropriate allocation of resources and expertise in higher-risk patients. Artificial intelligence driven technologies, risk prediction models, and intraoperative aids in surgery have been recently identified as an area for potential exponential growth over the next few years^60,61^. Despite mounting evidence of the substantial impact of human factors on the quality of performance in surgery and, subsequently, patient safety, the practical day-to-day significance of fatigue and burn-out remains overlooked^62^.

Male sex and the presentation with AC should be taken into account to inform the patient consenting process and shared decision-making on the timing of surgery, settings, surgical approach, and expertise required to perform the operation in the safest possible conditions.

## Supplementary Material

zraf076_Supplementary_Data

## Data Availability

All data analysed in this study were extracted from published papers and are available in the *Supplementary material*. The original datasets for these papers are not available for analysis.

## Supplementary materials

This includes supplementary results (patient age and comorbidities, timing of surgery as emergency cholecystectomy and laparoscopic *versus* open cholecystectomy) *Figs S1*, *S2* and *Table S1*. It also includes a spreadsheet with data extracted for all studies included in the analysis. *Supplementary material* is available at *BJS Open* online.
