# Supplementary material for: Risk factors and mitigating measures associated with bile duct injury during cholecystectomy: meta-analysis
Source: BJS Open. 2025 Aug 2;9(4):zraf076. doi: 10.1093/bjsopen/zraf076 (PMC12317273; doi:10.1093/bjsopen/zraf076)
Supplement: zraf076_Supplementary_Data [file zraf076_supplementary_data.zip › Supplementary_Material.docx]

Risk factors and mitigating measures associated with bile duct injury during cholecystectomy: meta-analysis

Rowan Burns^1^, Katie L Connor^1^, Rachel V. Guest^1^, Chris C. Johnston^1,2^, Ewen M. Harrison^1^,
Stephen J. Wigmore^1^, Ahmed E. Sherif^1,2,3^

^1^ Department of Clinical Surgery, University of Edinburgh, Royal Infirmary of Edinburgh, UK

^2^ Edinburgh Transplant Centre, Royal Infirmary of Edinburgh, UK

^3^ National Liver Institute, Menoufia University, Egypt

**Correspondence:**

Professor Stephen J Wigmore, Department of Clinical Surgery, University of Edinburgh, Royal Infirmary of Edinburgh, Edinburgh, United Kingdom EH16 4SA.
Email: s.wigmore@ed.ac.uk

**Supplementary Materials - Index**

| **Supplementary Results**  Patient age and co-morbidities  Timing of surgery as emergency cholecystectomy  Laparoscopic vs open cholecystectomy | *Page 2*  *Page 2*  *Page 2* |
| --- | --- |
| **Supplementary Figures and Tables** |  |
| **Figure S1.** Forest plots showing pooled unadjusted risk estimates for the relationship between the operating surgeon experience with the incidence of BDI. | *Page 4* |
| **Figure S2.** Forest plots showing pooled unadjusted and adjusted risk estimates (A and B) comparing laparoscopic vs open approach on the incidence of BDI. | *Page 4* |
| **Table S1.** Search terms used for all databases searched. | *Page 5* |
| **Supplementary References** | *Page 6* |
| **Supplementary data extraction spreadsheet** | *Attached separately* |

**Supplementary Results**

Patient age and co-morbidities

Eleven papers analysed age as a possible risk factor for BDI ([1-11](#_ENREF_1)). The patients’ age group comparisons were too heterogeneous for a pooled analysis. However, several studies did show in their analysis that patients older than 65 had higher odds ratios of BDI after cholecystectomy ([8-12](#_ENREF_8)).

Ten studies analysed co-morbidities in relation to BDI risk ([1](#_ENREF_1), [3-6](#_ENREF_3), [8](#_ENREF_8), [11](#_ENREF_11), [13-15](#_ENREF_13)). The co-morbidities were frequently reported utilising the Charlson Comorbidity Index to predict 10 years of survival in patients with multiple co-morbidities or the American Society of Anaesthesiology (ASA) score for classification of patient health before surgery ([16](#_ENREF_16), [17](#_ENREF_17)). None of the studies aimed to investigate the impact of comorbidities on BDI specifically, but few from population-based analyses found patients with multiple co-morbidities before surgery are possibly associated with increased incidence of BDI ([8](#_ENREF_8), [18](#_ENREF_18)). One study showed that morbid obesity as a comorbidity had a statistically significant impact on the incidence of BDI with adjusted OR [95% CI] = 2.8 [2.1–4.3] ([10](#_ENREF_10)).

Timing of surgery as emergency cholecystectomy

Twelve studies analysed the impact of the timing of surgery as an emergency on the risk of BDI development ([1](#_ENREF_1), [4](#_ENREF_4), [5](#_ENREF_5), [10](#_ENREF_10), [11](#_ENREF_11), [13](#_ENREF_13), [18-22](#_ENREF_18)). The timing of emergency surgery of the included cohort was defined as within 48 hours of emergency admission in one study ([10](#_ENREF_10)), while another sizeable population-based study described early cholecystectomy to have been within the first seven days of the index admission ([19](#_ENREF_19)). The remaining studies had undefined timing in distinguishing emergency from delayed elective cholecystectomy. Only two studies had well-defined timing of delayed surgery for acute cholecystitis ([19](#_ENREF_19), [22](#_ENREF_22)). At the same time, the remaining studies did not report the indication for the planned/elective cholecystectomies. One large population-based matched study found a link between delayed cholecystectomy for acute cholecystitis and increased incidence of BDI and prolonged post-operative hospital stay. Overall, combining the results of those studies was deemed inappropriate due to the significant population heterogeneity, precluding meaningful interpretations.

Laparoscopic vs open cholecystectomy

Eleven studies compared laparoscopic cholecystectomy (LC) with open approach (OC) in their analysis ([8](#_ENREF_8), [12](#_ENREF_12), [15](#_ENREF_15), [18](#_ENREF_18), [21-28](#_ENREF_21)). Seven studies had suitable data comparing both approaches, including one randomised clinical trial ([12](#_ENREF_12), [18](#_ENREF_18), [21](#_ENREF_21), [23-25](#_ENREF_23), [28](#_ENREF_28)). Both unadjusted and risk-adjusted analyses did not show any significant effect size favouring either approach (Figure S2). These were also limited by the considerable heterogeneity across included studies arising from the wide timescale for study inclusion (1977-2012), when laparoscopic cholecystectomy had evolved to have been the gold standard approach in acute and chronic benign indications for cholecystectomy. In the risk-adjusted pooled analysis, Fletcher et al., in their population-based study (1988-1994) from Australia, reported that the open approach is associated with a lower risk of BDI ([12](#_ENREF_12)). In contrast, Tornqvist et al. (201, in their national registry multivariable risk-adjusted analysis from Sweden (2005-2010), showed that the open approach was correlated with a significantly higher odds ratio of BDI (OR [95% CI] = 1.56 [1.26-1.94]) ([18](#_ENREF_18)). The only randomised controlled trial identified was by Kiviluoto et al ([25](#_ENREF_25)). It compared laparoscopic to open approaches for acute and gangrenous cholecystitis. The trial did not report any BDI events in either group and was therefore limited by the relatively small sample size (63 patients).

**Supplementary Figures and Tables**

**Figure S1.** Forest plots showing pooled unadjusted risk estimates for the relationship between the operating surgeon experience with the incidence of BDI.

**Figure S2.** Forest plots showing pooled unadjusted and adjusted risk estimates (A and B) comparing laparoscopic vs open approach on the incidence of BDI.

A)

B)

**Table S1.** Search terms used for all databases searched.

| Database | Search Terms |
| --- | --- |
| MEDLINE | "bile duct injury"[All Fields] OR "biliary injury"[All Fields]) AND ("Risk Factors"[Mesh] OR "outcomes"[All Fields] OR "outcome"[All Fields]) AND "cholecystectomy"[mesh] |
| EMBASE | (("bile duct injury" or "biliary injury") and ("Risk Factors" or "outcomes" or "outcome") and "cholecystectomy").tw. |
| Scopus | TITLE-ABS ("bile duct injury" OR  "biliary injury" )  AND  TITLE-ABS ( "Risk Factors"  OR  "outcomes"  OR  "outcome" )  AND  TITLE-ABS ( "cholecystectomy" )  AND  ( LIMIT-TO ( DOCTYPE ,  "ar" ) |
| Web of Science | ALL = (("bile duct injury" or "biliary injury") and ("Risk Factors" or "outcomes" or "outcome") and "cholecystectomy") |
| Cochrane CENTRAL | (("bile duct injury" or "biliary injury") and ("Risk Factors" or "outcomes" or "outcome") and "cholecystectomy") |

**Supplementary References**

1. Beliaev AM, Booth M. Late two-stage laparoscopic cholecystectomy is associated with an increased risk of major bile duct injury. Anz Journal of Surgery. 2016;86(1-2):63-8.

2. Fletcher E, Seabold E, Herzing K, Markert R, Gans A, Ekeh AP. Laparoscopic cholecystectomy in the Acute Care Surgery model: risk factors for complications. Trauma Surg Acute Care Open. 2019;4(1):e000312.

3. Flum DR, Dellinger EP, Cheadle A, Chan L, Koepsell T. Intraoperative Cholangiography and Risk of Common Bile Duct Injury during Cholecystectomy. JAMA. 2003;289(13):1639-44.

4. El-Dhuwaib Y, Slavin J, Corless DJ, Begaj I, Durkin D, Deakin M. Bile duct reconstruction following laparoscopic cholecystectomy in England. Surgical Endoscopy. 2016;30(8):3516-25.

5. Giger U, Ouaissi M, Schmitz SFH, Krahenbuhl S, Krahenbuhl L. Bile duct injury and use of cholangiography during laparoscopic cholecystectomy. British Journal of Surgery. 2011;98(3):391-6.

6. Lilley EJ, Scott JW, Jiang W, Krasnova A, Raol N, Changoor N, et al. Intraoperative cholangiography during cholecystectomy among hospitalized medicare beneficiaries with non-neoplastic biliary disease. American journal of surgery. 2017;214(4):682-6.

7. Yaghoubian A, Saltmarsh G, Rosing DK, Lewis RJ, Stabile BE, De Virgilio C. Decreased bile duct injury rate during laparoscopic cholecystectomy in the era of the 80-hour resident workweek. Archives of Surgery. 2008;143(9):847-51.

8. Tornqvist B, Stromberg C, Akre O, Enochsson L, Nilsson M. Selective intraoperative cholangiography and risk of bile duct injury during cholecystectomy. Br J Surg. 2015;102(8):952-8.

9. Altieri MS, Yang J, Obeid N, Zhu C, Talamini M, Pryor A. Increasing bile duct injury and decreasing utilization of intraoperative cholangiogram and common bile duct exploration over 14 years: an analysis of outcomes in New York State. Surgical Endoscopy. 2017;32(2):667-74.

10. Aziz H, Pandit V, Joseph B, Jie T, Ong E. Age and Obesity are Independent Predictors of Bile Duct Injuries in Patients Undergoing Laparoscopic Cholecystectomy. World journal of surgery. 2015;39(7):1804-8.

11. Dolan JP, Diggs BS, Sheppard BC, Hunter JG. Ten-year trend in the national volume of bile duct injuries requiring operative repair. Surgical Endoscopy. 2005;19(7):967-73.

12. Fletcher DR, Hobbs MST, Tan P, Valinsky LJ, Hockey RL, Pikora TJ, et al. Complications of cholecystectomy: Risks of the laparoscopic approach and protective effects of operative cholangiography - A population-based study. Annals of Surgery. 1999;229(4):449-57.

13. Kohn JF, Trenk A, Kuchta K, Lapin B, Denham W, Linn JG, et al. Characterization of common bile duct injury after laparoscopic cholecystectomy in a high-volume hospital system. Surgical Endoscopy. 2018;32(3):1184-91.

14. Ragulin-Coyne E, Witkowski ER, Chau ZL, Ng SC, Santry HP, Callery MP, et al. Is Routine Intraoperative Cholangiogram Necessary in the Twenty-First Century? A National View. Journal of Gastrointestinal Surgery. 2013;17(3):434-41.

15. Tornqvist B, Waage A, Zheng ZL, Ye WM, Nilsson M. Severity of Acute Cholecystitis and Risk of Iatrogenic Bile Duct Injury During Cholecystectomy, a Population-Based Case-Control Study. World Journal of Surgery. 2016;40(5):1060-7.

16. Charlson ME, Pompei P, Ales KL, MacKenzie CR. A new method of classifying prognostic comorbidity in longitudinal studies: development and validation. J Chronic Dis. 1987;40(5):373-83.

17. Saklad M. GRADING OF PATIENTS FOR SURGICAL PROCEDURES. Anesthesiology. 1941;2(3):281-4.

18. Tornqvist B, Waage A, Zheng ZL, Ye WM, Nilsson M. Severity of Acute Cholecystitis and Risk of Iatrogenic Bile Duct Injury During Cholecystectomy, a Population-Based Case-Control Study. World Journal of Surgery. 2016

;40(5):1060-7.

19. De Mestral C, Rotstein OD, Laupacis A, Hoch JS, Zagorski B, Alali AS, et al. Comparative operative outcomes of early and delayed cholecystectomy for acute cholecystitis: A population-based propensity score analysis. Annals of Surgery. 2014;259(1):10-5.

20. Kholdebarin R, Boetto J, Harnish JL, Urbach DR. Risk factors for bile duct injury during laparoscopic cholecystectomy: a case-control study. Surg Innov. 2008;15(2):114-9.

21. Natsume S, Kato T, Hiramatsu K, Shibata Y, Yoshihara M, Aoba T, et al. Presence of Aberrant Anatomy Is an Independent Predictor of Bile Duct Injury During Cholecystectomy. International surgery. 2017;102:250-7.

22. Ragulin-Coyne E, Witkowski ER, Chau Z, Ng SC, Santry HP, Callery MP, et al. Is Routine Intraoperative Cholangiogram Necessary in the Twenty-First Century? A National View. Journal of Gastrointestinal Surgery. 2013;17(3):434-42.

23. Diamantis T, Tsigris C, Kiriakopoulos A, Papalambros E, Bramis J, Michail P, et al. Bile duct injuries associated with laparoscopic and open cholecystectomy: An 11-year experience in one institute. Surgery Today. 2005;35(10):841-5.

24. Hobbs MS, Mai Q, Knuiman MW, Fletcher DR, Ridout SC. Surgeon experience and trends in intraoperative complications in laparoscopic cholecystectomy. Br J Surg. 2006;93(7):844-53.

25. Kiviluoto T, Siren J, Luukkonen P, Kivilaakso E. Randomised trial of laparoscopic versus open cholecystectomy for acute and gangrenous cholecystitis. The Lancet. 1998;351(9099):321-5.

26. Russell JC, Walsh SJ, Mattie AS, Lynch JT. Bile duct injuries, 1989-1993: A statewide experience. Archives of Surgery. 1996;131(4):382-8.

27. Shawhan RR, Porta CR, Bingham JR, McVay DP, Nelson DW, Causey MW, et al. Biliary Leak Rates After Cholecystectomy and Intraoperative Cholangiogram in Surgical Residency. Military Medicine. 2015;180(5):565-9.

28. Targarona EM, Marco C, Balague C, Rodriguez J, Cugat E, Hoyuela C, et al. How, when, and why bile duct injury occurs - A comparison between open and laparoscopic cholecystectomy. Surgical Endoscopy-Ultrasound and Interventional Techniques. 1998;12(4):322-6.
